# Transient paradoxical bronchospasm associated with inhalation of the LAMA AZD9164: analysis of two Phase I, randomised, double-blind, placebo-controlled studies

**DOI:** 10.1186/1471-2466-14-52

**Published:** 2014-03-27

**Authors:** Carin Jorup, Thomas Bengtsson, Kerstin Strandgården, Ulf Sjöbring

**Affiliations:** 1 AstraZeneca R&D Mölndal, Pepparedsleden 1, 431 83 Mölndal, Sweden; 2 StatMind AB, Scheelevägen 2 1, 223 81 Lund, Sweden

**Keywords:** LAMA, Muscarinic, Long-acting, COPD, Bronchodilator, Pharmacokinetics, Pharmacodynamics, Safety, Tolerability

## Abstract

**Background:**

AZD9164 has demonstrated potential as an inhaled, long-acting, muscarinic antagonist (LAMA) bronchodilator. However, in patients with COPD, but not in healthy subjects, a transient initial drop in FEV_1_ was observed following inhalation of nebulised doses of AZD9164 in citrate buffer.

Two additional studies were conducted to further assess the safety and tolerability of multiple ascending doses of AZD9164 in 27 white and 18 Japanese healthy subjects and in 4 patients with COPD. In these studies, AZD9164 was inhaled via Turbuhaler™.

**Methods:**

These were Phase I, randomised, double-blind, placebo-controlled, multiple ascending dose (MAD) studies conducted in Sweden and UK. Healthy subjects (mean age 25.9 yrs) and patients with COPD (mean age 66 yrs, mean post-bronchodilator FEV_1_ 60.1% predicted normal value) were randomised 2:1 to active treatment (400, 1000 or 2800 μg delivered doses of AZD9164) or placebo.

**Results:**

No safety or tolerability concerns were identified in the healthy subjects at doses up to and including 2800 μg and both studies confirmed the bronchodilator effect of AZD9164. However, the first 3 patients in the COPD cohort who received AZD9164 (1000 μg) experienced a transient fall in FEV_1_ 5 to 15 minutes after inhalation of AZD9164 while the patient receiving placebo did not. The study safety review process then resulted in cessation of further activities on AZD9164. Retrospective analysis showed that two healthy subjects had also had transient falls in FEV_1_ shortly after inhalation of AZD9164 400 and 2800 μg respectively, although neither reported any related respiratory symptoms or other AEs.

**Conclusions:**

These results show that transient paradoxical bronchoconstriction can occur in some healthy subjects, in addition to patients with COPD, following inhalation of AZD9164 and that the citrate buffer used in the nebulised formulation cannot have been the only cause of the drop in FEV_1_ in previous studies. As preclinical data do not provide an explanation, the reasons for this brief post-dose drop in FEV_1_ remain unclear. However, these results highlight the importance of monitoring lung function immediately post-dose when investigating novel inhaled treatments, even when a rapid onset of effect is not expected.

**Trial registration:**

Clinicaltrials.gov NCT01016951 and NCT01096563.

## Background

Chronic obstructive pulmonary disease (COPD), is globally a major and growing cause of morbidity and mortality [1,2]. COPD is characterised by progressive airflow limitation that is not fully reversible and is associated with neutrophil-mediated airway inflammation [3]. Current first line treatment options include long- and short-acting inhaled bronchodilators, which have little or no effect on the continuing decline in lung function. Monotherapy with inhaled glucocorticosteroid therapy is less effective in COPD than it is in asthma, which may be due to the differing inflammatory processes involved [1].

Long-acting bronchodilators, whether administered twice-daily, such as the β_2_-agonists formoterol and salmeterol and the recently approved muscarinic antagonist aclidinium, or once-daily, such as indacaterol, tiotropium and the recently approved olodaterol, vilanterol, glycopyrronium and umeclidinium, are the mainstay of COPD treatment. Other long-acting, once-daily bronchodilators are also in late stage development, including abediterol. The muscarinic antagonist tiotropium, in particular, has become well established as an effective once-daily bronchodilator for COPD over the past decade, with safety and efficacy demonstrated in large scale trials lasting up to three years. Tiotropium does, however, cause the anticholinergic side effect of dry mouth in 6–16% of patients with COPD [4-8]. With the aim of finding an alternative with an improved therapeutic index relative to tiotropium, a development program for a novel, long-acting, inhaled muscarinic antagonist (LAMA) bronchodilator for the treatment of patients with COPD was initiated.

The first compound from this program to be extensively evaluated in a clinical setting is AZD9164, a product of a collaboration between AstraZeneca Discovery and Pulmagen Therapeutics Limited (formerly Argenta Discovery Limited). *In vitro*, AZD9164 is a potent, competitive antagonist at the human, rat, guinea pig and dog muscarinic M_3_ receptor with similar potency at human M_1_, M_2_, M_4_ and M_5_ receptors. *In vivo*, AZD9164 is a potent antagonist against methacholine-induced bronchoconstriction in anaesthetised guinea pigs and has a much longer duration of action than ipratropium (>12 vs. 4 h) in this model. Preclinical safety pharmacology, pharmacokinetics (PK) and toxicology studies of AZD9164 revealed no significant adverse findings that would preclude studies in humans. AZD9164 demonstrated an improved therapeutic index in guinea pig models of lung (methacholine-induced bronchoconstriction) vs systemic (pilocarpine-induced salivation) muscarinic antagonist effects. These pre-clinical studies indicated that AZD9164 is a potent, long-acting muscarinic antagonist (LAMA), and suitable for further assessment in clinical studies.

An initial single ascending dose (SAD) study in 48 healthy human subjects (ClinicalTrials.gov Identifier NCT00847249) established the safety and tolerability of AZD9164 at lung deposited doses ranging from 4 to 1940 μg, and indicated a bronchodilating effect at the higher doses (≥700 μg). In a subsequent single dose crossover study in 28 patients with COPD, all tested doses of AZD9164 (100 μg, 400 μg and 1200 μg lung deposited doses) resulted in statistically significant increases in peak, trough and 24-h average FEV_1_ versus placebo [9]. The bronchodilator effect of AZD9164 400 μg was shown to be superior to tiotropium 18 μg in this study [9]. However, the study also indicated a transient initial, dose-dependent drop in FEV_1_ following nebulisation [9]. In both studies, AZD9164 was administered dissolved in citrate buffer and inhaled via nebulisation. The two studies described in this paper were conducted to further assess the safety and tolerability of AZD9164 after administration of multiple ascending doses (MAD) in healthy white subjects and in patients with COPD (NCT01016951; GMAD study) and in healthy Japanese subjects (NCT01096563; JMAD study). In these studies, AZD9164 was administered in the morning as a citrate-free dry powder via ^a^Turbuhaler™.

### Objectives

The present cohort with COPD patients was added to the original GMAD study protocol because the earlier COPD study had revealed a transient dose-dependent decrease in FEV_1_ that occurred 15 minutes after inhalation of AZD9164 but not after placebo or tiotropium. In contrast, no such effects had been seen in the single ascending dose (SAD) study in healthy subjects, which had involved even higher doses of AZD9164 and the same type of citrate buffer. However, the first FEV_1_ measurement was taken at 90 minutes post-dose in the SAD study, so that small transient drops in FEV_1_ that occurred before this time would not have been detected. The patients with COPD in the single-dose cross-over study were not exposed to the citrate buffer when given tiotropium or placebo. It was therefore unclear whether the decrease in FEV_1_ with AZD9164 was due to AZD9164 or to the citrate buffer in the nebuliser solution, the quantities of which increased in line with the dose of AZD9164. The present studies were designed to resolve these conflicting findings and to further assess the pharmacokinetic and pharmacodynamic profiles of AZD9164 by administering AZD9164 as a citrate-free dry powder via Turbuhaler™.

## Methods

### Study objectives

The primary objectives of the two MAD studies were to investigate the safety and tolerability of inhaled AZD9164 delivered via Turbuhaler™ in healthy white and Japanese subjects and also, in the GMAD study, in patients with COPD.

Secondary objectives of the studies were to characterise the pharmacokinetic (PK) profile and the pharmacodynamic (PD) effects of AZD9164 inhaled via Turbuhaler™ and to establish whether AZD9164 as a dry powder formulation is associated with bronchoconstriction in subjects with COPD. The COPD cohort was added to the original study protocol to provide additional data in support of this objective.

### Study designs

The GMAD study was conducted at two centres in Sweden, while the JMAD study was conducted at a single centre in the UK. Both were Phase I, randomised, double-blind, placebo-controlled studies. They were conducted according to the Declaration of Helsinki and Good Clinical Practice guidelines and the AstraZeneca policy on Bioethics. Independent ethics committees approved the study protocols, subject information and consent forms. The GMAD study was approved by the independent central Ethics Committee in Stockholm, Vetenskapsrådet, 103 78 Stockholm, Sweden. The JMAD study was approved by the Plymouth Independent Research Ethics Committee (PIREC), Tamar Science Park, Plymouth, UK. All subjects gave written informed consent. The first subject was enrolled in the GMAD study on 16 December 2009 and the last subject last visit was on 18 June 2010. In the JMAD study, the first subject was enrolled on 16 April 2010 and the last subject last visit was on 4 June 2010. Subjects were resident in the clinic from Day 1 to Day 17 of the study period.

**Figure 1:**
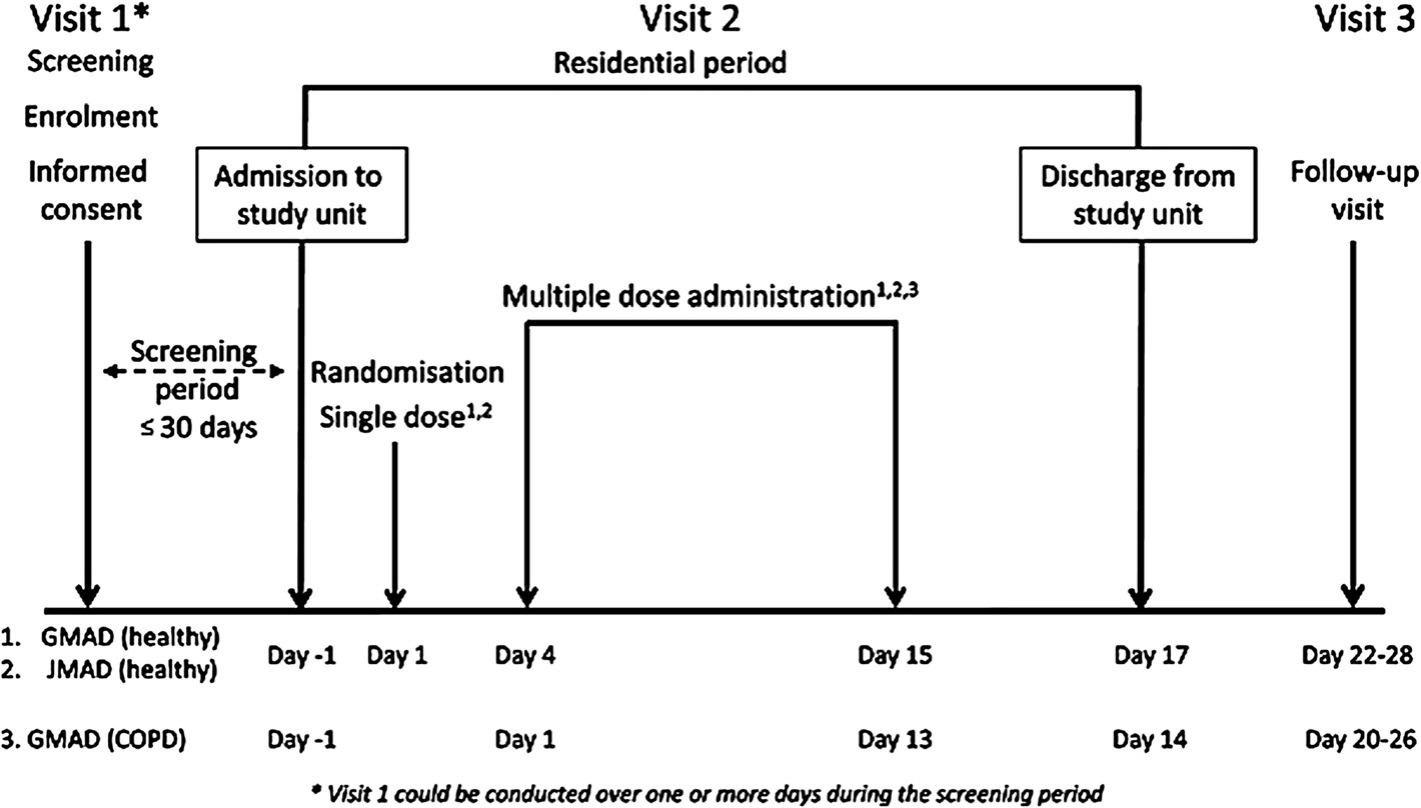
Flow chart of study designs – GMAD, JMAD and GMAD COPD cohort.

Due to the exploratory nature of these studies the sample sizes were not based on formal statistical considerations. The sample sizes were based on experience from previous similar Phase I studies with other compounds. In both MAD studies, the healthy subjects were randomised 2:1 to active treatment or placebo in three cohorts of 9 subjects. The randomisation scheme was produced by AstraZeneca R&D using the global randomisation system GRand. Subjects were allocated to AZD9164 or placebo using consecutive randomisation codes (subject numbers), so for cohorts 1 to 3, numbers began with 101, 201 and 301. The planned doses of AZD9164 were 400 μg delivered dose inhaled via Turbuhaler™ in cohort 1, 1000 μg in cohort 2 and 2800 μg in cohort 3. This equated to lung deposited doses of approximately 200, 600 and 1500 μg, according to *in vitro* batch testing of the Turbuhaler™ prior to starting the studies. Each subject received a single dose of AZD9164 or placebo on Day 1 and subsequent doses once daily between Day 4 and Day 15 (Figure 1). The initial single dose on Day 1 was followed by a wash-out period of 72 h to determine single-dose PK.

The studies were double-blind with regard to treatment (AZD9164 or placebo) at each dose level. Only the AstraZeneca personnel carrying out the labelling and packaging of study drug and analysing the PK samples had access to the randomisation list. Individual treatment codes, indicating the treatment randomisation for each randomised subject, were available to the investigators or pharmacists at the study centre. Individual sealed subject codes (one for each subject) with instructions for code breaking were provided to the Principal Investigators. The treatment code was not to be broken except in medical emergencies when the appropriate management of the subject required knowledge of the treatment randomisation. The Principal Investigators, after confirming eligibility and obtaining informed consent, ensured that each potential subject was assigned a unique enrolment number and a unique randomisation code (subject number). Study nurses primed all inhalers prior to first use.

After the last dose for each cohort, a Safety Review Committee (SRC) evaluated all available data in a blinded manner with the possibility of un-blinding if necessary, and based on this determined the subsequent dose. Each subject participated in 1 cohort only. The study design therefore allowed a gradual escalation of dose with intensive safety monitoring between each dose level to ensure the safety of the subjects. In both studies, a range of stopping criteria was pre-determined both for individual subjects/patients and for the study as a whole. These criteria included serious or non-tolerable adverse events, clinically significant changes in laboratory values or other safety parameters, pre-defined changes in cardiac function such as QTc prolongation (defined as QTcF > 500 ms, or an increase of QTcF >60 ms above baseline to a value >480 ms) and reaching pre-defined maximal exposure levels (total Cmax and/or AUC of 48 nM and/or 158 nM*h, respectively on day 15). In view of the fall in FEV_1_ seen in the previous study, the discontinuation criterion ‘Fall in FEV1 ≥ 30% compared with the pre-dose value on the same day within 4 h after administration of investigational product’ was added to the original protocol in relation to individuals within the study. The related overall study discontinuation criterion was ‘Two or more subjects, who receive AZD9164, have other clinically significant changes in laboratory values or other safety parameters’.

In another addition to the original protocol, nine COPD patients were to be randomised 2:1 to active treatment or placebo in cohort 4 of the GMAD study. These patients were to receive the 1000 μg mid dose given to healthy subjects for 13 consecutive days with no washout following the first dose (Figure 1).

### Study subjects

All subjects in these studies had to have the ability to use the Turbuhaler™ correctly and to provide informed consent and have veins suitable for repeated venepuncture or cannulation. All male subjects had to agree to use barrier contraception from the first day of dosing until 3 months after the last dose of study medication (no female subjects were enrolled except in the GMAD COPD cohort).

In the GMAD study, inclusion criteria for healthy subjects included age 18–45 years, body mass index (BMI) 18–30 kg/m^2^ and weight 50–100 kg. For the COPD cohort, male or female patients were to be aged over 40 years, with a BMI 18–32 kg/m^2^, weight 50–100 kg and to have had a clinical diagnosis of COPD (GOLD criteria) for > 1 year at enrolment, with a post-bronchodilator FEV_1_ 40–80% of the predicted normal value and post-bronchodilator FEV_1_/FVC <70%. All were to be current or ex-smokers with a smoking history of ≥10 pack years and to have had a recent (<9 months) chest radiograph showing no pathological changes that would make them unsuitable for inclusion. Female patients were to have negative pregnancy tests at screening and on admission to the unit and to have no child-bearing potential, as confirmed by investigator assessment during screening. In the Japanese study, inclusion criteria included age 20–45 years, BMI 18–27 kg/m^2^ and weight 50–85 kg. All of the subject’s parents and grandparents had to be Japanese and the subject must have been born in Japan, have a valid Japanese passport and have lived outside Japan for no more than 5 years.

Exclusion criteria for all studies included history of any clinically significant disease or disorder, or indications of such conditions or of drug or alcohol abuse during screening, that could put subjects or patients at risk by participation in the study or interfere with absorption, distribution, metabolism or excretion of drugs or otherwise affect study results. A family history or presence of glaucoma or symptoms of narrow-angle glaucoma such as headache, serious eye pain or halo phenomena in the evening or at night were also grounds for exclusion from the studies. Intake of grapefruit, Seville oranges or related products within 7 days of first administration of study medication was another exclusion criterion. For the COPD patients, a COPD exacerbation or treatment with systemic glucocorticosteroids (GCS) for any indication within 30 days of the first visit, history of arrhythmia, heart block or other ECG abnormalities, urinary retention or bladder neck obstruction or need for long-term oxygen therapy and/or saturation <92% were additional grounds for exclusion. With the exception of inhaled β_2_-agonists, use of medications that prolong the QT/QTc interval such as certain antihistamines, anti-arrhythmics, tricyclic antidepressants and monoamine oxidase inhibitors was not permitted for the COPD patients. Use of short-acting β_2_-agonists and use of inhaled corticosteroids (ICS) at a stable dose by the COPD patients was permitted during the study period. Use of long-acting β_2_-agonists, alone or in combination with inhaled corticosteroids and use of long-acting and short-acting muscarinic antagonists were not permitted.

The GMAD study was conducted by Quintiles at the Berzelius Clinical Research Centre, Linköping, Sweden and Quintiles Hermelinen, Luleå, Sweden, while the JMAD study was conducted by Richmond Pharmacology Ltd at St George’s University of London, UK.

**Figure 2:**
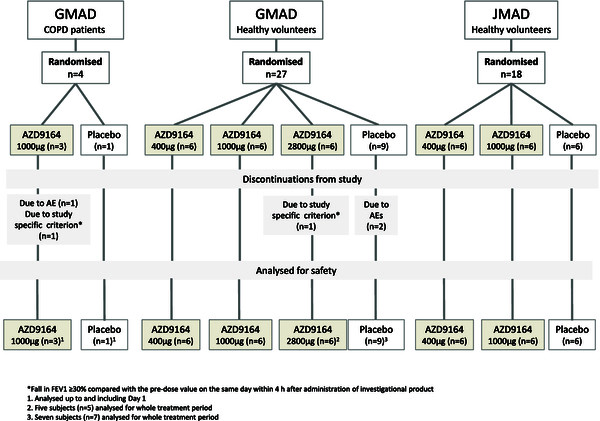
CONSORT patient flow diagram.

The demographic and key baseline characteristics of the healthy subjects and COPD patients are summarised in Table 1. Figure 2 is a CONSORT patient flow diagram for the studies.

**Table 1:** Study demographics

		**Placebo**	**AZD9164**			**Total**
**GMAD, healthy subjects**		**n = 9**	**400 μg n = 6**	**1000 μg n = 6**	**2800 μg n = 6**	**n = 27**
Age (yrs)	Mean	26	21	23	22	23
	SD	6	1	3	2	4
^a^BMI (kg/m^2^)	Mean	23.1	23.4	21.7	21.6	22.5
	SD	1.7	1.8	2.2	1.1	1.8
^a^Weight (kg)	Mean	73.8	80.3	70.7	70.0	73.7
	SD	6.8	8.1	7.4	4.7	7.6
**JMAD, healthy subjects**		**n = 6**	**400 μg n = 6**	**1000 μg n = 6**	**–**	**n = 18**
Age (years)	Mean	Mean 29.8	29.0	27.5	–	28.8
	SD	4.9	4.0	3.4	–	4.0
^a^BMI (kg/m^2^)	Mean	21.0	20.7	20.6	–	20.8
	SD	1.2	2.3	1.4	–	1.6
^a^Weight (kg)	Mean	61.3	63.6	61.4	–	62.1
	SD	4.3	7.7	5.5	–	5.8
**GMAD, COPD patients**		**n = 1**	**–**	**1000 μg n = 3**	**–**	**n = 4**
Age (yrs)	Mean	71	–	64	–	66
	SD	NC	–	7	–	6
^a^BMI (kg/m^2^)	Mean	23.0	–	27.8		26.6
	SD	NC	–	4.0		4.0
^a^Weight (kg)	Mean	77.1	–	73.9	–	74.7
	SD	NC	–	8.1	–	6.8
^b^FEV_1_ (L)	Mean	1.62	–	1.48	–	1.52
	SD	NC	–	0.53	–	0.43
^b^FEV_1_ (% predicted)	Mean	48.80	–	64.63	–	60.67
	SD	NC	–	17.82	–	16.57
^b^FEV_1_/FVC (%)	Mean	31.95	–	49.45	–	45.08
	SD	NC	–	5.66	–	9.90
Smoking history (%)	Current	1 (100)	–	2 (67)		3 (75)
	Former	0 (0)		1 (33)		1 (25)
Years since diagnosis	Mean	5		10		9
	SD	NC		9		8

^a^At enrolment.

^b^FEV1 and FVC measurements were made after inhalation of Atrovent® (post-bronchodilator).

*BMI* Body mass index, *NC* Not calculable.

### Materials

The investigational product was formulated at AstraZeneca R&D, Södertälje, Sweden and supplied as fully prepared individual Turbuhaler™s, requiring only priming prior to use. The dry powder for inhalation within each Turbuhaler™ was a mixture of AZD9164 and lactose monohydrate for inhalation. The placebo Turbuhaler™ was identical in appearance but contained lactose monohydrate powder only.

### Assessments

Safety assessments included recording of AEs, physical examination and changes in laboratory variables, vital signs (BP, pulse, body temperature), ECG and lung function (FEV_1_ and FVC). AEs were assessed throughout the treatment period and follow-up period. Blood samples were taken daily and telemonitoring was continuous during the residential period of the study. On Days 1 and 15, a 12-lead ECG was recorded at regular intervals from 30 minutes up to 48 h post-dose. Blood samples for PK measurements and laboratory values were taken pre-dose and at 5, 15, 30, 60 and 90 minutes and 2, 4, 6, 8, 12, 24, 36, 48, 72 and 120 h post-dose on Days 1 and 15. Pulse and BP were recorded pre-dose and at 30 min, 2, 4, 8, 12 and 24 h post-dose on days 1 and 15 and at 1 h post-dose on days 4 and 9. Lung function was recorded pre-dose and at 5, 15, 30 and 60 min and 2, 4, 12 and 24 h post-dose on Days 1 and 15, pre-dose and at 5, 15, 30 and 60 min and 2 h post-dose on days 4, 5 and 8, and pre-dose and at 15 min post-dose on days 6, 7 and 9–14 for the healthy subjects in the GMAD and JMAD studies. For the GMAD COPD cohort, lung function was to be recorded pre-dose and at 5, 15, 30 and 60 min and 2, 4, 12 and 24 h post-dose on days 1 and 13, pre-dose and at 5, 15, 30 and 60 min and 2 h post-dose on days 2, 3 and 6 and pre-dose and at 15 min post-dose on days 4, 5 and 7–12.

The PK profile of AZD9164 was assessed in terms of maximum plasma concentration (C_max_), time to reach C_max_ (t_max_), terminal half-life (t_1/2_), area under the plasma-concentration time curve (AUC; AUC_0–∞_ for first dose and AUC_0–24_ for last dose), dose-normalised C_max_ and AUC (C_max_/Dose, AUC/Dose) and accumulation ratio (R_ac_ = AUC_0–24_ for last dose/AUC_0–24_ for first dose).

The PD effects of AZD9164 were assessed by evaluating FEV_1_, FVC, supine BP, heart rate, pulse, QT interval with Fridericia’s correction (QTcF) over the first 4 h following first and last drug administration respectively (Days 1 and 15). Average effect (E_av_) and maximum effect (E_max_) over the first 4 h were calculated and recorded for all PD parameters except diastolic BP, for which E_av_ and minimum effect (E_min_) were calculated and recorded. Lung function (FEV_1_) was assessed from 0–4 h post-dose to ensure that E_max_ was recorded.

All statistical and PK analyses were performed by Quintiles AB, Global Phase I Services, Uppsala, Sweden. All statistical calculations were performed with the SAS® software version 9.2. Pharmacokinetic parameters were derived using standard non-compartmental methods with WinNonlin® Professional Version 5.2. The safety analysis sets included all subjects who received at least one dose of randomised investigational product and had data collected post-dose. The PK analysis sets included all subjects who received at least 1 dose of AZD9164 and from whom evaluable PK data appropriate for the analysis of interest were available. The PD analysis sets included all subjects who received at least 1 dose of randomised investigational product and for whom evaluable PD data appropriate for the analysis of interest were available. The as-treated principle was applied to all evaluations.

## Results

In the GMAD study, a total of 27 white healthy male subjects, aged 19 to 39 years, were randomised; all but three completed the study and received 13 administrations of AZD9164 or placebo. Of the three discontinuations, two subjects had received placebo and one had received AZD9164. One male and 3 female patients with COPD were included in a separate cohort. In the JMAD study, 18 Japanese healthy male subjects, aged 24–38 years, were randomised. All subjects in the first two cohorts completed the study and received all doses according to the study plan but the study was halted before the third cohort received the highest planned dose level of 2800 μg. Overall, the treatment groups were well balanced and comparable with respect to demographic characteristics.

### Safety evaluation

There were no safety or tolerability concerns identified in healthy subjects at doses up to and including 2800 μg.

In the GMAD study, there were no deaths recorded, but there were two discontinuations due to AE (DAE); both occurred in the placebo group. One was due to a serious adverse event (SAE), a brief asymptomatic ventricular tachycardia, which occurred on Day 5 almost 3 h after dose administration. The other DAE (due to pyrexia and diarrhoea) occurred on Day 10, between 11 and 13 h post-dose. Calicivirus RNA was subsequently detected in a faeces sample from this subject.

In the GMAD study, a total of 40 AEs was reported by the 18 healthy subjects who received AZD9164, with 31 AEs reported by the 9 who received placebo. The frequency of AEs was therefore greatest in the placebo-treated subjects. The frequency of AEs was lowest in the group receiving the lowest dose of AZD9164. A summary of adverse events is provided in Tables 2, 3 and 4. No AE with severe intensity was recorded. Seven AEs in the placebo group and 1 AE in the 1000 μg and 2800 μg group respectively were regarded to be of moderate intensity. The rest were of mild intensity. The seven AEs reported by three subjects that were assessed as causally related to treatment with AZD9164 included dry mouth (1 event), throat irritation (3 events), cough (2 events) and flushing (1 event). These were all mild in intensity and occurred only with the highest dose. Six healthy subjects receiving AZD9164 and one subject who received placebo reported respiratory-related AEs of which the most common was throat irritation (Table 3). Five of these respiratory-related AEs (throat irritation: 3 events and cough: 2 events) were reported by 2 subjects in the highest dose group (2800 μg). All of these events occurred in close association with either first or second dose and were assessed as causally related to treatment by the investigator. In the JMAD study, there were no deaths, SAEs or DAEs reported. All AEs were of mild intensity. Thirteen of the 18 Japanese subjects reported 20 adverse events after receiving AZD9164 or placebo. Six of these subjects received the 400 μg dose and four received the 1000 μg dose. There was no increase in adverse events or in subjects reporting AEs with increasing doses. Four of the AEs were considered to be causally related to the study drug in each of the 400 μg and 1000 μg dose cohorts. The most common AE was localised rash, associated with the ECG tabs in three of the six cases. Two subjects reported the respiratory AE of cough following treatment with AZD9164 on Day 1. One of these subjects also had an associated transient 11% decrease in FEV_1_ at five and 15 minutes after dosing (400 μg), and the other had a 20% reduction in FEV_1_ at five minutes post-dose (1000 μg). Both subjects coughed immediately after inhalation.

**Table 2:** Summary of frequency of adverse events across the studies

	**Placebo**	**AZD9164**			
**GMAD, healthy subjects**	**n = 9**	**400 μg**	**1000 μg**	**2800 μg**	**Total (%)**
			**n = 6**	**n = 6**	**n = 6**	**n = 27**
Any AE^a^	8 (88.9)	5 (83.3)	6 (100)	6 (100)	25 (92.6)
SAEs^a^	1(11.1)	0 (0)	0 (0)	0 (0)	1 (3.7)
DAEs^a^	2 (22.2)	0 (0)	0 (0)	0 (0)	2 (7.4)
Total number of AEs	31	7	18	15	71
**JMAD, healthy subjects**	**n = 6**	**400 μg**	**1000 μg**		**Total (%)**
		**n = 6**	**n = 6**		**n = 18**
Any AE^a^	3 (50.0)	6 (100.0)	4 (66.7)		13 (72.2)
SAEs^a^	0 (0)	0 (0)	0 (0)		0 (0)
DAEs^a^	0 (0)	0 (0)	0 (0)		0 (0)
Total number of AEs	3	10	7		20
**GMAD, COPD patients**	**n = 1**		**n = 3**		**n = 4**
Any AE^a^	1 (100)		3 (100)		4 (100)
SAEs^a^	0 (0)		0 (0)		0 (0)
DAEs^a^	0 (0)		1 (33.3)		1 (25.0)
Total number of AEs	1		8		9

^a^Number (%) of patients who had an AE in each category. Subjects with multiple events in the same category are counted only once in each category.

**Table 3:** Summary of patients affected by respiratory-related adverse events

**AEs classified as Respiratory, Thoracic and/or Mediastinal Disorders**					
	**Placebo**	**AZD9164**			
**GMAD, healthy subjects**	**n = 9**	**400 μg**	**1000 μg**	**2800 μg**	**Total (%)**
		**n = 6**	**n = 6**	**n = 6**	**n = 27**
Throat irritation	1 (11.1)	0 (0)	1 (16.7)	2 (33.3)	4 (14.8)
Oropharyngeal pain	0 (0)	1 (16.7)	2 (33.3)	0 (0)	3 (11.1)
Cough	0 (0)	0 (0)	0 (0)	1 (16.7)	1 (3.7)
Dyspnoea	0 (0)	0 (0)	1 (16.7)	0 (0)	1 (3.7)
**JMAD, healthy subjects**	**n = 6**	**400 μg**	**1000 μg**		**Total (%)**
		**n = 6**	**n = 6**		**n = 18**
Cough	0 (0)	1 (16.7)	1 (16.7)		2 (11.1)
Epistaxis	0 (0)	0 (0)	1 (16.7)		1 (5.6)
Nasal mucosal disorder	0 (0)	0 (0)	1 (16.7)		1 (5.6)
Respiratory tract infection	0 (0)	0 (0)	1 (16.7)		1 (5.6)
Upper respiratory tract infection	0 (0)	1 (16.7)	0 (0)		1 (5.6)
**GMAD, COPD patients**	**n = 1**		**n = 3**		**n = 4**
Dyspnoea	1 (100)		2 (66.7)		2 (50)
Cough	0 (0)		1 (33.3)		1 (25)

**Table 4:** Summary of adverse events causally related to treatment with AZD9164

	**AEs assessed as causally-related to AZD9164**				
	**Placebo**	**AZD9164**			
**GMAD, healthy subjects**	**n = 9**	**400 μg**	**1000 μg**	**2800 μg**	**Total (%)**
		**n = 6**	**n = 6**	**n = 6**	**n = 27**
Dry mouth	0			1 (16.7)	1 (3.7)
Throat irritation	0			3 (50.0)	3 (11.1)
Cough	0			2 (33.3)	2 (7.4)
Flushing	0			1 (16.7)	1 (3.7)
**JMAD, healthy subjects**	**n = 6**	**400 μg**	**1000 μg**		**Total (%)**
		**n = 6**	**n = 6**		**n = 18**
Rash	0	1 (16.7)			1 (5.6)
Headache	0	1 (16.7)			1 (5.6)
Cough	0	1 (16.7)	1 (16.7)		2 (11.1)
Upper respiratory tract infection	0	1 (16.7)	0		1 (5.6)
Respiratory tract irritation	0		1 (16.7)		1 (5.6)
Epistaxis	0		1 (16.7)		1 (5.6)
Nasal mucosal disorder	0		1 (16.7)		1 (5.6)
**GMAD, COPD patients**	**n = 1**		**n = 3**		**n = 4**
Dry mouth	0		1 (33.3)		1 (25.0)
Palpitations	0		1 (33.3)		1 (25.0)
Anxiety	0		1 (33.3)		1 (25.0)
Dyspnoea	0		2 (66.6)		2 (50.0)
Cough	0		1 (33.3)		1 (25.0)
Haematoma	0		1 (33.3)		1 (25.0)
Fatigue	0		1 (33.3)		1 (25.0)

There were no clinically important patterns in laboratory test results, vital signs or ECG parameters with increasing doses of AZD9164. Of particular interest, given the known potential for muscarinic antagonists to affect the QT interval, there were no QTcF values exceeding 450 ms and no notable changes from baseline in QTcF after administration of AZD9164. There were no marked differences between healthy subjects on active treatment and healthy subjects receiving placebo in either of the studies. There was a mean increase of 8 mmHg in average systolic blood pressure at the 2800 μg dose compared to placebo after the last dose in the GMAD study and a minor mean increase in diastolic blood pressure at the 1000 μg dose compared to placebo in the JMAD study.

### Effects on FEV_1_

Both studies provided further evidence of the bronchodilating effect of AZD9164. In the GMAD study, numerical mean increases vs placebo of up to 10% in the FEV_1_ E_max_ were seen in the healthy subjects. The increases were greater after repeated dosing, reaching statistical significance after the last dose of 2800 μg. In the JMAD study, there was a statistically significant improvement in both average and peak FEV_1_ over 4 h compared to placebo after the last doses of 400 μg and 1000 μg.

In the GMAD study, small, transient decreases in mean FEV_1_ were observed on Day 1 in healthy subjects at 15 minutes after administration of single doses of 400 μg AZD9164 (2.3% decrease), 2800 μg AZD9164 (5.5% decrease) and placebo (3.9% decrease) but not after administration of 1000 μg AZD9164 (1.4% increase). One healthy subject was discontinued from the GMAD study in accordance with the FEV_1_ discontinuation criterion (“a fall in FEV_1_ ≥ 30% compared with the pre-dose value within 4 h after administration of investigational product”). The maximum reduction in FEV_1_ (34%) for this subject occurred 30 minutes after the first dose of AZD9164 2800 μg on Day 1. This subject had no respiratory symptoms or any other AEs in connection with the fall in FEV_1_ and his FVC remained normal. The subject had an unusual exhalatory technique that resulted in difficulty in exhaling fully and correctly into the spirometer and his result may therefore have been an artefact. A second healthy subject in the GMAD study had a similar drop in FEV_1_ (31%) five minutes after the 6th dose of AZD9164 400 μg. The event was not associated with any respiratory symptoms or other AEs. A reporting error led to this subject’s fall in FEV_1_ being overlooked at the time, but the correct values were relocated retrospectively. Although there was no clear dose-dependency for the decrease in FEV_1_, the highest dose produced the largest of the individual falls recorded.

**Figure 3:**
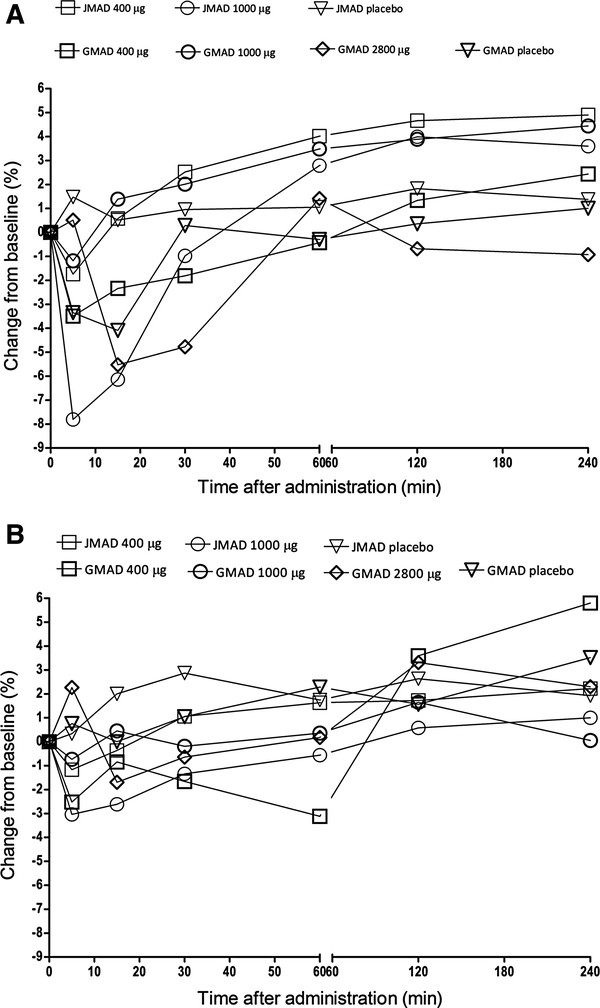
**Effects of AZD9164 on mean values for FEV**_**1 **_**over the first 4 h after dosing. A.** FEV_1_ 0–4 h after first dose – GMAD, JMAD combined data from healthy subjects. **B.** FEV_1_ 0–4 h after last dose – GMAD, JMAD combined data from healthy subjects.

In the JMAD study, a dose dependent initial mean decrease in FEV_1_ of 2% and 8% was observed at five minutes following dosing with AZD9164 400 μg and 1000 μg, respectively on Day 1 (Figure 3A). Five out of six subjects showed an initial decrease in FEV_1_ of between 4 and 20% in the 1000 μg group. However, increases above baseline FEV_1_ value were observed at 15 minutes (400 μg) and 1 hour (1000 μg) post-dose. Mean values for FEV_1_ 0–4 h in healthy subjects from the GMAD and JMAD studies after the first and last doses are shown in Figures 3A and 3B respectively.

### Pharmacokinetics

In healthy subjects, the absorption of AZD9164 from the lung was rapid and similar between doses and study populations with a median t_max_ of between 5 and 15 minutes. After C_max_, the plasma concentration-time profiles showed a multi-phasic, mainly parallel decline with a geometric mean t_½_ after the last dose of 166 h in the GMAD study and 118 h in the JMAD study. The plasma concentrations of AZD9164 were above the lower limit of quantification (LLOQ of 10.0 pmol/L) throughout the sampling periods i.e. 72 h after single dose and 120 h after last dose. The geometric mean R_ac_ after once daily dosing was 3.08 in the GMAD study and 2.77 in the JMAD study. A summary of relevant pharmacokinetic data is provided in Table 5.

**Table 5:** Summary of pharmacokinetic parameters for AZD9164 after single dose and after last dose (400 μg, 1000 and 2800 μg)

	**First dose**			**Last dose**		
**Geometric mean (CV%)**						
**PK parameters AZD9164**	**400 μg**	**1000 μg**	**2800 μg**	**400 μg**	**1000 μg**	**2800 μg**
	**(n = 6)**	**(n = 6)**	**(n = 6)**	**(n = 6)**	**(n = 6)**	**(n = 5)**
**GMAD Study healthy subjects**						
C_max_ (nmol/L)	1.32 (52.9)	3.93 (64.9)	13.3 (136.0)	1.95 (36.2)	7.47 (50.5)	16.0 (140.0)
t_max_ (min)^a^	10.0 (5–31)	10.0 (5–16)	5.0 (5–15)	11.0 (5–31)	5.0 (5–16)	5.0 (5–6)
t_½_ (h)	95.1 (19.5)	137 (41.1)	72.3 (58.5)	189 (62.4)	179 (86.1)	131.0 (55.9)
AUC^b^	11.8 (30.1)	41.3 (62.1)	54.5 (113)	12.1 (25.0)	39.4 (42.2)	58.6 (87.8)
C_max_/dose(10-3/L)	1.53 (52.9)	1.83 (64.9)	2.21 (136.0)	2.28 (36.2)	3.47 (50.5)	2.67 (140.0)
AUC/Dose^b^	0.0138 (30.1)	0.0193 (62.1)	0.0092 (113)	0.0141 (25.0)	0.0184 (42.2)	0.0098 (87.8)
R_ac_	-	-	-	3.23 (22.1)	3.52 (48.4)	2.47 (30.0)
**JMAD Study healthy subjects**						
C_max_ (nmol/L)	1.14 (30.8)	7.27 (47.6)	-	1.83 (33.2)	6.30 (31.8)	-
t_max_ (min)^a^	15 (5–60)	5 (5–5)	-	15 (5–90)	5 (5–15)	-
t_½_ (h)	88.1 (29.7)	71.3 (11.9)	-	107.3 (26.2)	130.2 (56.9)	-
AUC^b^	12.5 (23.0)	29.4 (49.3)	-	15.6 (18.7)	28.6 (40.9)	-
C_max_/dose(10-3/L)	1.33 (30.8)	3.39 (47.6)	-	2.13 (33.2)	2.93 (31.8)	-
AUC/Dose^b^	0.0145 (23.0)	0.0137 (49.3)	-	0.0182 (18.7)	0.0133 (40.9)	-
R_ac_	-	-	-	3.69 (24.4)	2.08 (25.1)	-

^a^Median (minimum to maximum).

^b^Zero to infinity for single dose; 0–24 for last dose.

There was no indication of dose dependency in PK in the 400 μg to 2800 μg delivered dose range in the GMAD study. No firm conclusion could be reached regarding dose dependency in the JMAD study (only two cohorts were completed, at doses of 400 μg and 1000 μg of AZD9164).

Plasma samples for pharmacokinetic analysis were collected from three COPD patients at 1 h, and from two of these patients at 24 h, following inhalation of a single dose of 1000 μg AZD9164. The plasma concentrations at 1 h were higher in COPD patients compared to those in healthy subjects; however the levels were within one doubling dose (assuming dose linearity). The concentrations at 24 h in COPD patients were similar to those of the healthy subjects.

### Evaluations for patients with COPD

The GMAD study was prematurely stopped after three COPD patients had been exposed to a single dose of AZD9164 (1000 μg) and one to placebo in the fourth cohort. The safety review process enabled unblinding of data for these patients which then led to the cessation of further study activities on AZD9164.

**Figure 4:**
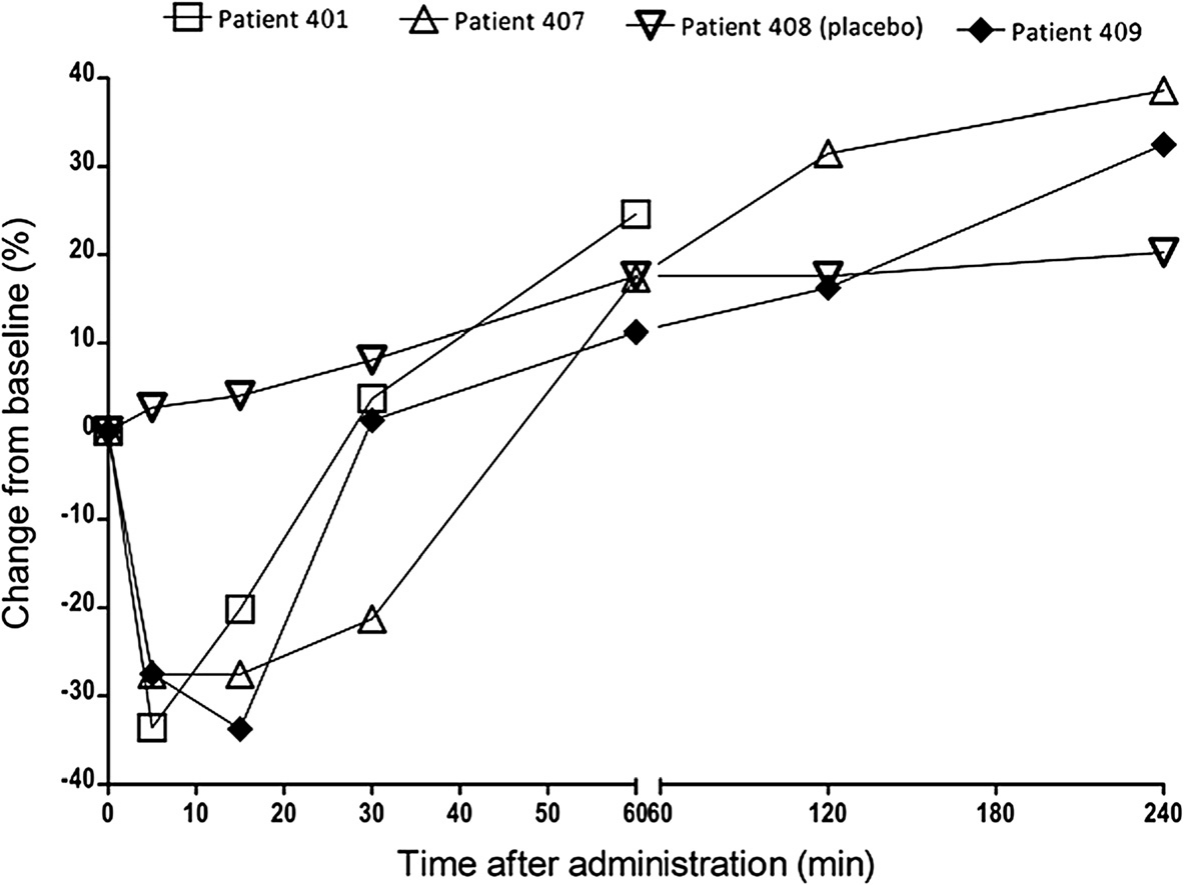
**FEV**_**1 **_**0–4 h after initial dose – GMAD study COPD patients.**

The three COPD patients who received the 1000 μg dose of AZD9164 experienced a fall in FEV_1_ of 34%, 28% and 34%, respectively, 5 to 15 minutes after inhalation of the first dose whereas no fall was noted for the patient receiving placebo. (Figure 4) Two of these patients also reported mild dyspnoea but had no other clinically relevant symptoms. The patient with a fall in FEV_1_ of 34% also experienced a DAE, due to severe anxiety, palpitations of moderate intensity and dry mouth of mild intensity. These symptoms were coincident with the fall in FEV_1_ and were assessed as causally related to study drug. In all three patients receiving AZD9164, however, FEV_1_ had returned to baseline or above within one hour and remained above baseline values at all subsequent time points to 4 h post dose. However, as two of the patients had met their individual FEV_1_ discontinuation criterion, the study discontinuation criterion “Two or more subjects, who receive AZD9164, have other clinically significant changes in laboratory values or other safety parameters” was also met.

In total, there was 1 DAE and 8 AEs among the three COPD patients who received a single dose of AZD9164 1000 μg in the GMAD study (Table 2). Three of the AEs were mild respiratory symptoms (2 dyspnoea, 1 cough). These were assessed by the investigator as being causally related to the study drug. The patient who received placebo also complained of dyspnoea but this was assessed as unrelated to treatment. Subsequent analysis revealed no clinically important abnormalities in laboratory safety test results, ECG measurements or vital signs assessments relating to these AEs.

## Discussion

The results of the small exploratory studies reported here indicate that the fall in FEV_1_ observed in COPD patients in the crossover study [9] was not due to the citrate content of the buffer alone. All three COPD patients who received citrate-free AZD9164 via Turbuhaler™ experienced a significant fall in FEV_1_ 5 to 15 minutes after inhalation of AZD9164, while the patient receiving placebo did not. Two of 18 healthy white subjects and 0 of 12 healthy Japanese subjects also demonstrated transient falls in FEV_1_ > 30% shortly after inhalation of AZD9164. Adverse events of a mainly respiratory nature were associated with the falls in FEV_1_, primarily in the COPD cohort. The development of AZD9164 for COPD has now been discontinued, due to these findings.

Despite preclinical assessment and subsequent re-evaluation in the light of these findings, the reason for the brief post-dose drop in FEV_1_ in some healthy subjects and all of the patients with COPD remains unclear. The M_1_, M_2_ and M_3_ receptors that have been demonstrated in human airways appear to have different physiological functions [10-12] The M_1_ receptors in airway parasympathetic ganglia facilitate cholinergic neurotransmission, which therefore indirectly enhances cholinergic bronchoconstriction. The M_3_ receptors on airway smooth muscle cells, inflammatory cells and submucosal glands directly mediate bronchoconstriction and mucus secretion. The M_2_ receptors at prejunctional cholinergic nerve endings control the release of acetylcholine and act as feedback inhibitory receptors. One explanation for the current findings could be that either partial agonist activity at the M_1_ receptor or M_2_ antagonist effects could hypothetically enhance cholinergic drive before the onset of M_3_ antagonism to inhibit the immediate increase in cholinergic drive. However, there was no indication from available preclinical data that this is the explanation for the observations with AZD9164. An additional, non-pharmacological explanation could be that AZD9164 has a localised irritant effect which is greater in the inflamed lung tissue of patients with COPD than in healthy subjects.

Paradoxical bronchospasm after inhalation of a bronchodilator is not a novel occurrence. A review of adverse reaction reports for inhaled β_2_-selective, adrenergic-agonist bronchodilators between 1974 and 1988 revealed 126 reports consistent with a diagnosis of paradoxical bronchospasm associated with use of these drugs by metered-dose inhaler, and a further 58 such reports between 1983 and 1988 for these drugs delivered by nebulisation [13]. Salbutamol has been shown to cause occupational asthma in pharmaceutical workers and has an asthma hazard index of 0.84 on a scale of 0–1, with a value of 0.5 or higher indicating asthmagenic potential [14].

Paradoxical bronchoconstriction may cause a fall in FEV_1_ with no other symptoms or it may cause cough, wheeze and dyspnoea and the need for use of rescue medication. It may be caused by an adverse reaction to the drug being administered or to the excipients, preservatives, surfactants, stabilisers or propellants used in its delivery [15-18]. Abrupt changes in osmolarity, temperature or pH in lung tissue resulting from inhalation of a drug aerosol, airflow turbulence, deep inhalation or expiration and repeated spirometry efforts may also trigger bronchospasm [16,19,20]. Which of these factors was involved in these studies is unclear, but the dual response to AZD9164 raises interesting scientific issues which will continue to be explored.

## Conclusions

The initial bronchoconstrictor effect observed with AZD9164 makes this molecule unfit for its intended purpose. However, the experience in early clinical evaluation of AZD9164 has highlighted the importance of including early timepoint measurements of lung function when investigating novel inhaled treatments, even when a rapid onset of effect is not anticipated.

## Endnote

^a^Turbuhaler™ is a trademark of the AstraZeneca group of companies. Turbuhaler™ is a registered trademark in Sweden and United Kingdom where the studies were performed.

## Abbreviations

AUC: Area under the plasma concentration time curve; BP: Blood pressure; Cmax: Maximum plasma concentration; COPD: Chronic obstructive pulmonary disease; DBP: Diastolic blood pressure; Eav: Average pharmacodynamic effect; Emin: Minimum pharmacodynamic effect; Emax: Maximum pharmacodynamic effect; FEV1: Forced expiratory volume in one second; FVC: Forced vital capacity; GMAD: Global multiple ascending dose study; JMAD: Japanese multiple ascending dose study; LABA: Long-acting β_2_-agonist; LAMA: Long-acting muscarinic antagonist; LLOQ: Lower limit of quantification; QTcF: QT interval with Fridericia’s correction for heart rate; Rac: Accumulation ratio; SBP: Systolic blood pressure; t½: Terminal half life; tmax: Time to maximal plasma concentration (C_max_).

## Competing interests

CJ and US are employees of AstraZeneca and hold shares in the company. TB and KS were employees of AstraZeneca at the time this work was carried out. None of the authors has any other competing interest.

## Authors’ contributions

CJ was the AstraZeneca physician responsible for AZD9164. TB participated in the design of the study and performed the statistical analysis. KS was responsible for the pharmacokinetic analysis. US was a safety physician in the studies and participated in evaluation of safety-related data during conduct of both studies. All of the authors helped to draft the manuscript and read and approved the final manuscript.

## Pre-publication history

The pre-publication history for this paper can be accessed here:

http://www.biomedcentral.com/1471-2466/14/52/prepub

## References

[B1] Barnes PJ Chronic obstructive pulmonary disease: a growing but neglected global epidemic PLoS Med 2007 4 e112 10.1371/journal.pmed.0040112 17503959 PMC1865560

[B2] Pauwels R Rabe KF Burden and clinical features of chronic obstructive pulmonary disease (COPD) Lancet 2004 364 9434 613 620 10.1016/S0140-6736(04)16855-4 15313363

[B3] Barnes PJ Shapiro SD Pauwels RA Chronic obstructive pulmonary disease: molecular and cellular mechanisms Eur Respir J 2003 22 672 688 10.1183/09031936.03.00040703 14582923

[B4] Vogelmeier C Hederer B Glaab T Schmidt H Rutten-van Mölken M Beeh KM Rabe KF Fabbri LM Tiotropium versus salmeterol for the prevention of exacerbations of COPD N Engl J Med 2011 364 1093 1103 10.1056/NEJMoa1008378 21428765

[B5] Yohannes AM Willgoss TG Vestbo J Tiotropium for treatment of stable COPD: a meta-analysis of clinically relevant outcomes Respir Care 2011 56 477 487 10.4187/respcare.00852 21255503

[B6] Santus P Di Marco F Safety and pharmacological profile of tiotropium bromide Expert Opin Drug Saf 2009 8 387 395 10.1517/14740330902953684 19505267

[B7] Tashkin DP Impact of tiotropium on the course of moderate-to-very severe chronic obstructive pulmonary disease: the UPLIFT trial Expert Rev Respir Med 2010 4 279 289 10.1586/ers.10.23 20524910

[B8] ZuWallack AR ZuWallack RL Tiotropium bromide, a new, once-daily inhaled anticholinergic bronchodilator for chronic-obstructive pulmonary disease Expert Opin Pharmacother 2004 5 1827 1835 10.1517/14656566.5.8.1827 15264997

[B9] Bjermer L Bengtsson T Jorup C Lötvall J Local and systemic effects of inhaled AZD9164 compared with tiotropium in patients with COPD Respir Med 2012 107 84 90 23098686 10.1016/j.rmed.2012.09.014

[B10] Barnes PJ The pharmacological properties of tiotropium Chest 2000 117 63S 66S 10.1378/chest.117.2_suppl.63S 10673478

[B11] Fryer DA Jacoby DB Muscarinic Receptors and Control of Airway Smooth Muscle Am J Respir Crit Care Med 1998 158 S154 S160 10.1164/ajrccm.158.supplement_2.13tac120 9817739

[B12] Belmonte KE Cholinergic pathways in the lungs and anticholinergic therapy for chronic obstructive pulmonary disease Proc Am Thorac Soc 2005 2 297 304 10.1513/pats.200504-043SR 16267352

[B13] Nicklas RA Paradoxical bronchospasm associated with the use of inhaled beta agonists J Allergy Clin Immunol 1990 85 959 964 10.1016/0091-6749(90)90084-H 1970585

[B14] Seed MJ Agius RM Paradoxical asthma hazard of short-acting b2-agonists Allergy 2008 63 241 18028244 10.1111/j.1398-9995.2007.01576.x

[B15] Beasley CRW Rafferty P Holgate ST Bronchoconstrictor properties of preservatives in ipratropium bromide (Atrovent) nebuliser solution Brit Med J 1987 294 1197 1198 2954609 10.1136/bmj.294.6581.1197-a PMC1246359

[B16] Hodder R Pavia D Lee A Bateman E Lack of paradoxical bronchoconstriction after administration of tiotropium via Respimat® Soft Mist™ Inhaler in COPD Int J COPD 2011 6 245 251 10.2147/COPD.S16094 PMC3144844 21814460

[B17] Mutlu GM Moonjelly E Chan L Olopade CO Laryngospasm and paradoxical bronchoconstriction after repeated doses of beta 2-agonists containing edetate disodium Mayo Clin Proc 2000 75 285 287 10725956

[B18] Gupta P O’Mahoney MS Potential adverse effects of bronchodilators in the treatment of airways obstruction in older people: recommendations for prescribing Drugs Aging 2008 25 415 443 10.2165/00002512-200825050-00005 18447405

[B19] Suzuki S Miyashita A Matsumoto Y Okubo T Bronchoconstriction induced by spirometric manoeuvres in patients with bronchial asthma Ann Allergy 1990 65 315 320 2145792

[B20] Israel RH Kohan JM Poe RH Kallay MC Greenblatt DW Rathbun S Inhaled metaproterenol is superior to inhaled cromolyn in protecting against cold-air-induced bronchospasm Respiration 1988 53 225 231 10.1159/000195426 3140327

